# Evaluation of the sealer/gutta-percha ratio on sets of root section surfaces of some extracted teeth sealed using the cold lateral condensation technique

**DOI:** 10.25122/jml-2021-0102

**Published:** 2021

**Authors:** Ioana Suciu, Bogdan Dimitriu, Mihai Ciocardel, Mihaela Chirila, Oana Amza, Sinziana Scarlatescu, Cristina Preoteasa, Mihaela Grigorie, Monica Voiculeanu

**Affiliations:** 1. Faculty of Dental Medicine, Carol Davila University of Medicine and Pharmacy, Bucharest, Romania; 2. Department of Geology, Petroleum-Gas University of Ploiesti, Ploiesti, Romania

**Keywords:** filling techniques, cold lateral condensation, gutta-percha-filled area

## Abstract

Canal filling must be well adapted to the walls of the root canal to prevent bacterial infiltration. Endodontic seals play an essential role in ensuring tightness, without which the canal filling would suffer infiltrations. This study aimed to evaluate the areas occupied by the two components of the canal filling, as well as the sealer/gutta-percha ratio in the root canals of the maxillary central incisors after their filling using the cold lateral condensation technique with gutta-percha. Thirty extracted upper central incisors were rotatably prepared with ProTaper Universal up to F3 and sealed using the cold lateral condensation technique with gutta-percha. After setting the sealer, the roots of the teeth were sectioned perpendicularly to 1 (L1), 3 (L3), 6 (L6), and 8 (L8) mm from the apex. The surface of the sections was analyzed with a Leica EZ4D stereomicroscope and photographed at two magnification orders: 10x and 25x. The areas corresponding to the gutta-percha, sealer, gaps, and root canal were expressed in pixels using the ImageJ software, version 1.50i. The difference in the representation of sealer areas, gutta-percha and voids was statistically significantly different for all four sections analyzed. The best adaptation of the canal obturation was observed in L1 and L3. The gutta-percha area was statistically significantly higher than that of the sealer for the L1, L3, and L6 levels, while the sealer/gutta-percha ratio recorded the lowest value at the L3 level (0, 30) and the highest at its L8 (0.70) level, without registering statistically significant differences regarding the area at the four analyzed levels. The voids were mostly absent or recorded a minimal percentage area (<1%). Cold lateral condensation of gutta-percha has led to a good adaptation of gutta-percha to the root canal wall, with a small amount of sealer, especially to the sections made at 3 mm from the apex. Given the limitations of this study, we noted that the voids were few – observed in the 6 and 8 mm sections – and were negligible in many cases.

## INTRODUCTION

A good canal filling must prevent the infiltration of bacteria and their degradation products along the root canal, up to the apical region. There are various methods to achieve the sealing of the root canals, from lateral condensation and single cones to thermoplastic techniques. The root canal should be filled mainly with a non-resorbable material such as gutta-percha, along with a small amount of resorbable material such as a sealer.

The presence of the sealer in the root canal prepared from a mechanical point of view, in the case of gutta-percha filling techniques, has the role of filling the microscopic spaces between the gutta-percha and the walls of the root canal. Endodontic sealants play an important role in preventing infiltration of canal fillings [1, 2].

Ideally, the sealer should be present as small as possible and be located between the dentinal root surface and the main non-absorbable core material due to the inability of the latter to fill all surfaces and irregularities of the root canal. Infiltrations of the root canals may be due to the solubilization of the sealers over time, so keeping the sealer in a thin layer around a solid mass of gutta-percha is always preferable [3].

The cold lateral condensation technique remains one of the most used today with the following advantages: compared to the old technique of filling with paste and single cone, lateral condensation fills the spaces around the master cone with accessory cones, intimately sealed between them and between the root canal wall sealing cement; compared to the hot injection techniques of gutta-percha, where the plasticized gutta-percha adapts very well to the irregularities of the endodontic space, lateral condensation offers a less efficient result, especially when the canals have recesses, isthmuses and fins [4]. However, in oval canals, lateral condensation fails to fill the spaces beyond the action of mechanical tools for the preparation and widening of root canals [2].

Currently, the most appreciated sealers have an increased ability to penetrate the dentinal tubules and the ability to bind both dentin and the main filling material. Such sealants are those based on epoxy resins; among the most well-known and used is AH Plus (Dentsply, Sirona). Epoxy resin sealants were introduced by Schroeder [5] due to their low solubility, excellent apical sealing, and root retention in dentin [6]. Various ways of applying sealers have also been developed, such as the gun-based system of injection and two-component syringes, which improve not only the mixing of the sealer components but also the final properties of the sealing cement.

The commonly accepted ideal for canal fillings is a maximum volume of core material and a minimum amount of sealer. Previous studies have measured the areas employed by gutta-percha and sealer in cross-sections along the root in order to assess the quality of the filling canal [1, 2, 7–12]. In the present study, we aimed to evaluate the surfaces occupied by the two components of the root canal filling and the sealer/gutta-percha ratio in the root canals after their filling using the cold lateral condensation of the gutta-percha.

## MATERIAL AND METHODS

Teeth extracted in the surgery clinic of the Faculty of Dentistry within the Carol Davila University of Medicine and Pharmacy, Bucharest, Romania, were used for this purpose. Extractions were in all cases due to severely affected and complicated periodontal status after repeated and complex attempts to be treated in terms of periodontal issues. Periodontists from the Faculty of Dentistry were repeatedly consulted for almost all cases. Most extractions were done very easily, without any complications, due to the advanced mobility associated with the severe reduction of bone implantation. More than 60 teeth were obtained in this way, but we decided to include in the study only single-rooted teeth due to the known difficulties that may occur in the case of detailed post-extraction investigations of multi-rooted teeth.

Forty human maxillary central incisors were collected and stored in distilled water. The teeth had been extracted for periodontal reasons, unrelated to this study. Preoperative radiographs were taken from both proximal and buccal views to ensure a patent, single and straight canal. The roots were inspected for cracks, developmental anomalies, resorption, fractures, and teeth with such defects were discarded.

The access cavity was prepared with an F 801/018 diamond round bur (Dia Tessin, Switzerland). The canal length was measured using a 10 k-file until its tip was visible at the apical foramen at surgical microscope (SmartOPTIC, Seliga microscopes, Poland). The working length was established by subtracting 1 mm from this length. The glide path was created with K-file 10–15 (FKG Dentaire, Switzerland).

All the root canals were instrumented using the ProTaper Universal system (Dentsply Sirona, Switzerland) up to the F3 instrument, size 30.09 taper. The endodontic engine was set at 250 rpm with 2 Ncm torque. The canals were prepared in a crown-down manner, beginning with an SX file in the coronal part of the canal. S1 and S2 instruments were used with brushing motions first in the two-thirds of the canal and then at the entire working length. F1, F2 and F3 instruments were used with in-and-out pecking motions until the full working length was reached.

Between each instrument, the canals were irrigated with 2 ml of 3% NaOCl (Parcan, Septodont, France) using a syringe and a 28-gauge Max-I-Probe needle that was placed 1 mm short from the working length. The smear layer and dentin debris produced during instrumentation were removed using 3 ml of 17% EDTA (Cerkamed, Poland) for 1 minute. The root canals were finally flushed with 5 ml distilled water and dried using paper points. The canal walls were lightly coated with AH Plus (Dentsply Sirona) sealer, using a K file by pumping and counterclockwise movements.

### Root canal filling: the lateral condensation technique

A 30.02 taper gutta-percha cone was trimmed to have tug back at the working length. The apical tip of the master gutta-percha cone was coated with sealer and placed in the canal followed by insertion of a 30 size finger spreader 1 mm short from the working length, which was maintained for 20 seconds, then rotated and withdrawn. An accessory gutta-percha cone, coated with a sealer, was placed in the space provided by the spreader, and the process was repeated, pressing both cones against the canal wall. The procedures were repeated until the spreader could not penetrate more than 2–3 mm into the root canal. The gutta-percha cones were cut with a hot plugger and condensed with a cold one (P. Machtou, no. 4, Dentsply Sirona, Switzerland). The filled roots were stored at 37°C and 100% humidity for seven days to allow the complete set of the sealer.

In order to appreciate the sealer/gutta-percha ratio, extracted single-rooted teeth with canal fillings were sectioned perpendicular to the long axis, obtaining root section surfaces. For this purpose, a microtome was used to obtain most sections (Smart Cut 6010, UKAM Industrial Superhard Tools, Valencia, CA USA).

**Figure 1:**
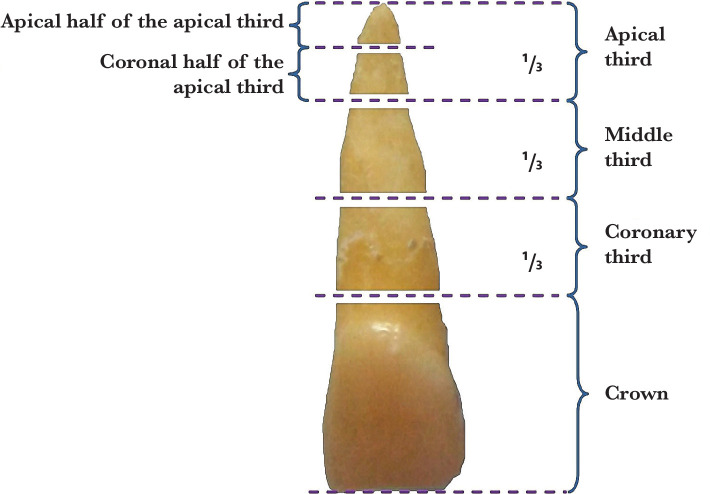
Illustration of the sectioning of the teeth roots: in the apical third at 1 and 3 mm, in the middle third at 6 mm, in the coronary third at 8 mm.

The roots of the teeth were sectioned into four segments as follows: in the apical third at 1 mm from the apex (marked L1) and at 3 mm from the apex (marked L3), in the middle third at 6 mm from the apex (marked L6) and the coronal third at 8 mm from the apex (noted L8) [13]. This sectioning method is schematically represented in Figure 1.

The coronal surface of each root segment was photographed with a Leica EZ4D laboratory research stereomicroscope (Leica Microsystems, Germany), with a built-in digital camera, at two magnification levels: 10x and 25x. When shooting at the 25x magnification order, the aim was to focus the root canal in the microscope field.

Each digital image obtained had a resolution of 2048×1536 pixels and was computer-marked using the original software of the device, with a standard size. On the 25x, digital images were also scored relatively precisely; the components of interest in the endodontic area were the sealer, gutta-percha, and any voids where appropriate.

After microscopic examination of the root section surfaces, other teeth, besides the multi-rooted ones, were removed from the study. The reasons have been mainly thin breaking segments of the roots, most likely following existing cracks and in some cases, the presence of a sealer with abnormal consistency, which has led to unfavorable appearance changes in the sectional area (perhaps the sealer was part of a two-component system and the activator has been used in an adequate proportion). Thus, in the end, only 30 upper central incisors were operated on.

An unmarked duplicate set of images at the magnification order of 25x was used separately for computerized digital analysis. A specialized software (ImageJ, version 1.50i) was used to determine the areas corresponding to gutta-percha, sealer, and any gaps on the surface section. The areas were initially expressed in pixels. The absolute values of the areas in mm^2^ were not calculated since we considered that they are not significant for establishing the sealer/gutta-percha ratio for each section surface. The proportions of the endodontic space occupied by the sealer and gutta-percha, at each level at which the section surface was made, were expressed by reporting the number of pixels in the digital image corresponding to the respective component of the canal filling to the total number of pixels corresponding to the endodontic space (area of the root canal in pixels).

### Statistical analysis

The statistical analysis emphasized the representation of the areas of the sealer, gutta-percha and gaps; in each section analyzed, the nonparametric Kruskal Wallis test was used. The groups were statistically different (p<0.05), and post-hoc analysis was performed with Bonferroni correction. Thus, for the Bonferroni correction, the areas of gutta-percha and sealer were compared with the Mann-Whitney test, using a statistical significance threshold of 0.0125, calculated by dividing p=0.05 to the compared number of groups, namely the 4 levels analyzed (p<0.05/4 = 0.0125). We used the nonparametric Kruskal Wallis test to investigate whether there are differences between the areas of sealer, gutta-percha, voids for each of the 4 sections analyzed.

The Statistical Package for the Social Sciences (SPSS) software was used for statistical analysis. The considered statistical significance threshold was p<0.005, with the above remarks.

## RESULTS

### Representation of sealer and gutta-percha areas reported at the section level

At all levels of the section, gutta-percha recorded the most significant area, as a percentage, followed by the sealer area. The difference in the representation of the sealer areas, gutta-percha and voids was statistically significantly different for all the four sections analyzed (Kruskal Wallis test, p<0.001). According to the Mann-Whitney test results, the gutta-percha area was statistically significantly larger than the sealer area for the L1 (p=0.001), L3 (p=0.001), and L6 (p=0.001) levels. The difference at the L8 level was not statistically significant (p=0.017).

The images were grouped as plates of two columns of 4. On the left column, there are the images seen at a 10x magnification, completely revealing the section area. On the right column, there are the images seen at a 25x magnification, which do not necessarily cover the entire surface of the root section but are centered on the root canal. Each row in a plate corresponds to one of the coronal-apical sectioning levels.

**Figure 2:**
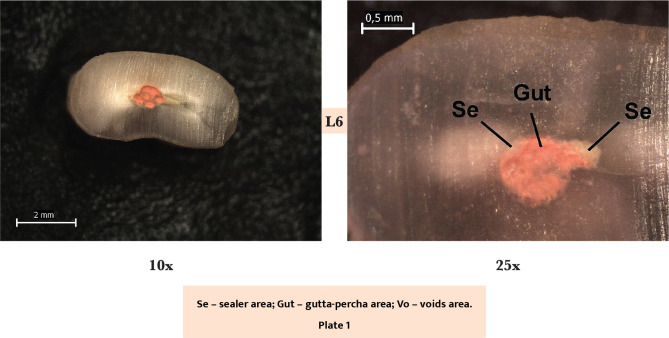

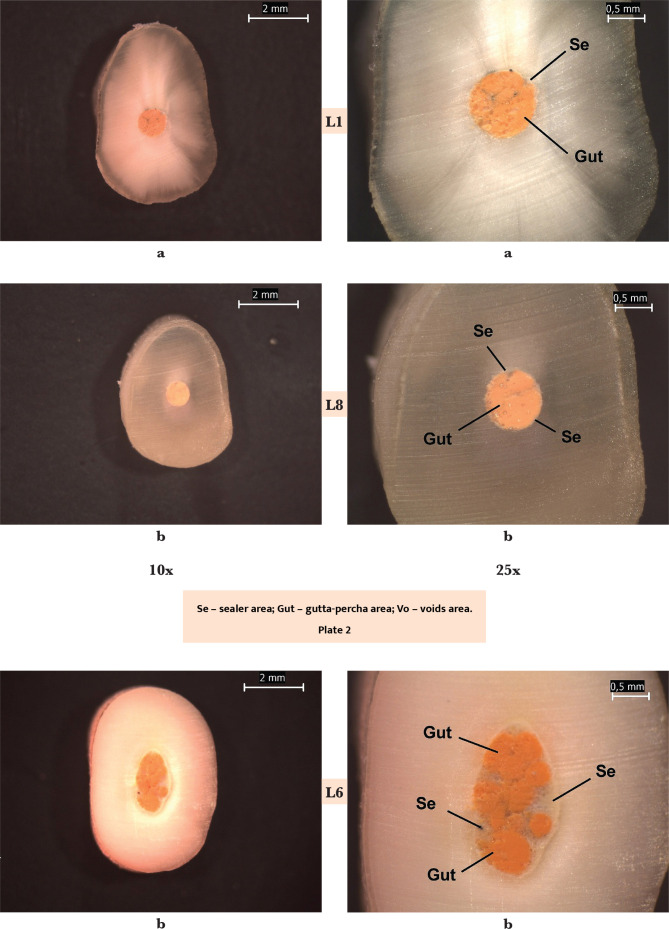

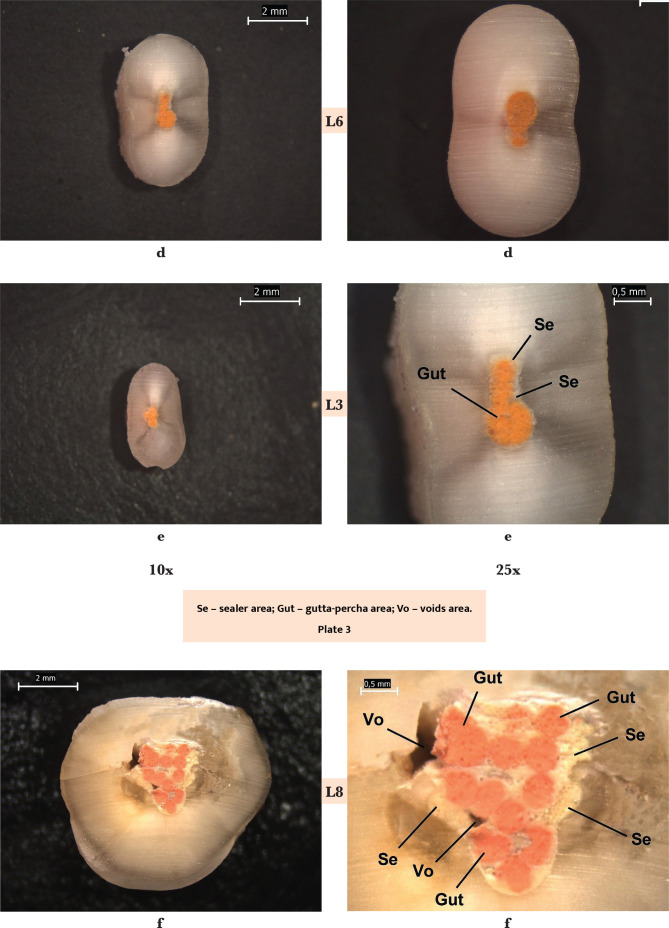

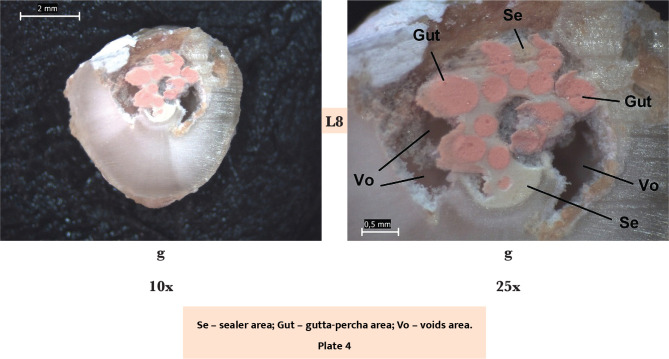
Section surfaces evaluted by stereomicroscopy.

Most of the sections at the L1 level showed a uniform and regular mass of gutta-percha. The percentage of gutta-percha varied between 55.11% and 85.34%, with an average of 69.83, whereas the average percentage of sealer was 25.48 (Table 1). Also, at L3, a good adaptation of gutta-percha to the root canal wall was observed, and the sealer was in small quantity, covering the walls circumferentially, with an average of 26.76 (Figure 2: plates 1–3, A–E). The highest percentage of gutta-percha was recorded at that level, ranging from 92.37% to 63.89%, with an average of 77.51. Moreover, the lowest percentage sealer/gutta-percha (0.30) was registered here as well.

**Table 1:** The percentage results of sealer, gutta-percha, voids and sealer/gutta-percha ratio in the 1, 3, 6 and 8 mm sections.

Aspect analyzed	Level	Mean	Median	Minimum	Maximum
**Sealer**	L1	25.48	27.09	14.65	32.17
	L3	26.76	29.35	16.28	37,62
	L6	30,90	31.70	17.31	49.65
	L8	36.88	34.91	3.57	61.52
**Gutta-percha**	L1	69.83	69.11	55.11	85.34
	L3	77.51	80.63	63.89	92.37
	L6	63.36	65.69	35.95	82.69
	L8	62.25	61.38	38.47	96.42
**Voids**	L1	0.08	0	0	0.60
	L3	0.14	0	0	0.50
	L6	3.67	0	0	24.71
	L8	0.86	0	0	6.07
**Sealer/gutta-percha**	L1	0.38	0.44	0.17	0.49
	L3	0.30	0.24	0.08	0.56
	L6	0.58	0.52	0.20	1.38
	L8	0.70	0.53	0.03	1.59

In almost half of the L6 sections, the root canal fillings were well adapted to the canal walls (Figure 2, plate 3, CD). In the other half, gutta-percha cones surrounded by a thicker layer of sealer and even sealer that penetrated the irregularities of the endodontic space (Figure 2: plates 1 and 3, CD) were observed. The sealer/gutta-percha ratio was increased (0.58).

L8 sections showed, in most cases, insulated gutta-percha cones with sealer around and sealer areas inside the gutta-percha mass (Figure 2: plates 1 and 3, CD). In some cases, a better adaptation of the gutta-percha was observed, with small amounts of sealer peripherally covering the canal (Figure 2, plate 2, AB). The highest sealer/gutta-percha ratio (0.70) was recorded here.

### Percentage area of sealer/gutta-percha/voids reported at the section level

Regarding the sealer, its area was the smallest at the L1 level and the largest at the L8 level. However, it did not register statistically significant differences regarding the area at the four analyzed levels – Kruskal Wallis test χ 2 (3)=4.84, p=0.184. The gutta-percha area was the smallest at the L8 level and the largest at the L3 level. However, it did not register statistically significant differences regarding the area at the four analyzed levels – Kruskal Wallis test χ 2 (3)=5.19, p=0.158.

Regarding the voids, their area was the smallest at the L1 level and the largest at the L6 level. However, there were no statistically significant differences regarding the area at the four analyzed levels – Kruskal Wallis test χ 2 (3)=1.49, p=0.683. The gaps were mostly absent or recorded a very small percentage area (<1%). In 2 specimens (L6 and L8), we observed 2 larger voids of 24.71% and 6.07%, respectively, of the occupied surface (Figure 2: plate 4, FG). The percentage of other voids was very small overall, with most values being 0 or close to 0, so it can be ignored [14].

## DISCUSSION

Although previous studies have used the radiography method to analyze the quality of root canal fillings [15, 16], Goldberg *et al*. have shown that orolingual radiographs provide limited information and that mesiodistal incidences are more suitable for the analysis of canal fillings [17]. However, it is quite challenging to differentiate gutta-percha from sealer on radiographs. Therefore, radiographs are unsuitable for the correct assessment of the quality of canal fillings and any resulting gaps. The literature has shown that root sectioning can correctly assess root canal obturation at different levels [12, 18, 19]. In the present study, the method of sections and observations under the stereomicroscope was used, which provides a real image of the canal fillings, and the images were analyzed using the ImageJ software, version 1.50i. The sections were made using the microtome so that during sectioning, the heat released would not distort the filling material.

The apical region is biologically important because it emphasizes the apical closure. This is alsothe reason why two sections were made at this level – 1 and 3 mm. If there is a large amount of sealer here, it can contract or dissolve over time [1, 20]. In the sections made at this level, a good adaptation of the sealer and gutta-percha to the root canal walls was noticed. A possible explanation is that the penetration of the spreader up to 1 mm length along the cone of gutta-percha, which significantly improves the homogeneity of the root canal filling [21–23]. A further step should be to remove the smear layer effectively, using a syringe with a Max-I-Probe needle, brought up to within 1 mm of the working length.

In contrast to our study, Eguchi *et al*. have reported that the lowest percentage level of gutta-percha at 2 and 4 mm from the apex (78.4% and 80.8%, respectively) was observed after using the lateral condensation method, indicating a large amount of sealer [7]. Also, Soo *et al*. reported the most sections (made at 1 and 3 mm) with irregular gutta-percha masses with voids and sealer areas on the perimeter of the channels after the lateral condensation of the C-shaped oval channels [13]. Gencoglu *et al*. showed that vertical condensation techniques led to a higher gutta-percha/sealer ratio than that obtained by lateral condensation [10], while Farea *et al*. concluded in their study that the sealer area decreases towards the coronal portion, while the gutta-percha area increases towards the coronal area [24].

The highest percentage of gutta-percha (77.51 %) and the lowest sealer/gutta-percha ratio (0.30) were found at 3 mm, and this can be explained by the fact that the oval channels tend to become round towards the apical area, and the filling will adapt better to the walls of the root canal. By comparison, De Deus *et al*. obtained higher percentages of gutta-percha in the sections made at 2 mm (89.94%) and 4 mm (87.57%), but they did not use sealer in the fillings of the root canal [12].

In the middle and coronal portions (L6 and L8), we observed the highest values of the sealer/gutta-percha ratio. These results are in agreement with those obtained by Marciano *et al*. [25] and Anbu *et al*. [26]. This may be due to the irregular shape and the larger volume of the canals in the coronal and mean third area, which leads to the mismatch of the tapered instrument with which it widens or fills the canal in these areas. One of the factors that can influence the result of canal filling can be correlated with the anatomical variability of the teeth used. Oval canals can be difficult to clean and fill [27, 28]. Lateral condensation has been and is still widely used in plugging root canals, but the results may differ in canals with different shapes and sizes [29]. At 3 mm from the apex, the uninstrumented recesses occur in a proportion of 45%, and at 5 mm from the apex, these insufficiently prepared recesses occur in a proportion of 65% [28]. The areas unaffected by the action of endodontic preparation instruments in irregularly shaped canals may frequently remain unobstructed when using the lateral condensation technique [28]. These statements correlate with the results of this study. The higher amount of sealer observed may be due to the inability of the lateral condensation technique to allow a homogeneous layer of sealer along the entire root canal [6, 30]. However, the clinical significance of the 30% or more sealer filling in the coronal or middle portion is unknown [14].

Also, the poor quality of the root canal filling at these levels might be due to unsatisfactory compaction of a greater number of accessory cones due to the large diameter of the incisors’ canals in these portions. The master cone used was 30.02 taper; thus, the apical adaptation was very good, but the spreader did not compact well the main cone and the accessory cones due to the increased section area in L6 and L8.

In the present study, we obtained few voids. Very small voids were found in only a few sections, and they could have gone unnoticed on x-rays. Only two specimens had larger voids between the dentinal wall and the filling material. Similar results were obtained by Anbu *et al*. [26]. The proper interfacial adapting may be due to the fact that AH Plus is correlated with increased adhesion to dentin and gutta-percha [6].

Regarding the sealing of oval channels, although some authors argue that these canals can be sealed properly by cold lateral condensation [31], most of them believe that thermoplasticized and vertically condensed gutta-percha is better suited to oval canal walls than cold gutta-percha cones, especially in the case of debridement and correct removal of the smear layer [14, 32–34]. In the case of wide, irregularly shaped channels, cold lateral condensation will result in a rather more flawed canal filling because the gutta-percha cones are somewhat displaced laterally instead of being compacted and deformed by the action of the spreader [28].

Lateral condensation is the most widely accepted and used technique of root canal filling [35, 36]. Although lateral condensation is easy to achieve and offers good apical sealing, it requires a large number of cones but also a longer procession time. It has also been associated with a lack of adaptation of gutta-percha to the root wall, with large amounts of sealer and the appearance of voids [7, 37]. Lateral condensation does not produce a homogeneous mass and may leave voids between the gutta-percha and the dentinal wall or accessory cones. According to Schilder *et al*., numerous cones are tightly pressed and joined together by the sealer, but the sealer can resorb and form voids in the lateral condensation technique [38].

## CONCLUSION

Cold lateral condensation of gutta-percha has led to a good adaptation of gutta-percha to the root canal wall, with a small amount of sealer, especially in the sections made at 3 mm from the apex. On the other hand, the highest ratio of sealer/gutta-percha was obtained in the coronal portion at 8 mm. Given the limitations of this study, the voids were few (recorded in the 6 and 8 mm sections) but were rather negligible in many cases.
